# Comparative Analysis of Growth Traits and Metabolic Profiles in *Camassia* Cultivars ‘Alba’ and ‘Caerulea’ Under Varying Cultivation Conditions

**DOI:** 10.3390/molecules30234520

**Published:** 2025-11-23

**Authors:** Alina-Ştefana Ozarchevici, Ilian Badjakov, Petko Mladenov, Ivayla Dincheva, Bogdan-Ionel Cioroiu, Lucia Draghia

**Affiliations:** 1 Faculty of Horticulture, Iasi University of Life Sciences “Ion Ionescu de la Brad”, Mihail Sadoveanu Alley No 3, 700490 Iasi, Romania; alina.ozarchevici@iuls.ro (A.-Ş.O.); lucia.draghia@iuls.ro (L.D.); 2 Department of Agrobiotechnologies, Agrobioinstitute, Agricultural Academy, 8 Dragan Tsankov Blvd., 1164 Sofia, Bulgaria; ibadjakov@abi.bg; 3 Department of Functional Genetics, Biotic and Abiotic Stress, Agrobioinstitute, Agricultural Academy, 8 Dragan Tsankov Blvd., 1164 Sofia, Bulgaria; rubisko@abv.bg; 4 Centre of Competence “Sustainable Utilization of Bio-Resources and Waste of Medicinal and Aromatic Plants for Innovative Bioactive Products” (BIORESOURCES BG), 8 Dragan Tsankov Blvd., 1164 Sofia, Bulgaria; 5 Research Centre for Oenology, Romanian Academy, Iași Branch, Mihail Sadoveanu Alley No 9, 700490 Iasi, Romania; bogdan.cioroiu@acadiasi.ro

**Keywords:** *Camassia leichtlinii*, bulbous geophytes, morphological traits, soil water balance, environmental adaptation, GC–MS metabolomics, primary metabolism

## Abstract

This study examines the morphological growth and metabolic responses of two *Camassia leichtlinii* cultivars, ‘Alba’ and ‘Caerulea’, cultivated under three contrasting systems: open field, outdoor pots, and greenhouse (indoor pots). Morphological parameters, including leaf number, scape development, and bulb biometric traits, were assessed over two consecutive growth seasons. Parallel GC–MS metabolite profiling identified 38 major compounds encompassing sugars, fatty acids, amino acids, and organic acids. Principal component analysis (PCA) and hierarchical clustering (HCA) effectively discriminated samples by cultivation condition, cultivar, and plant maturity. Environmental factors accounted for the largest share of metabolic variation (61%), followed by genotype (28%) and plant age (6%). The cultivar ‘Caerulea’ exhibited greater biomass accumulation and broader metabolic variability under greenhouse conditions, while ‘Alba’ maintained consistently high sucrose and glutamine levels across environments. Notably, the greenhouse environment, although strongly promoting primary metabolite accumulation, suppressed scape initiation and flowering in both cultivars, indicating a trade-off between metabolic enhancement and reproductive development under controlled conditions. These findings highlight differential adaptive strategies among *Camassia* cultivars and provide molecular insights into their carbohydrate metabolism, environmental responsiveness, and potential nutritional and phytochemical applications under diverse horticultural conditions.

## 1. Introduction

The genus *Camassia* (*Asparagaceae*) comprises six perennial taxa indigenous to North America, most notably *C. quamash* (Pursh) Green and *C. leichtlinii* (Baker) S. Watson, which are distributed widely across the Pacific Northwest and southwestern Canada. Members of this genus are appreciated for their ornamental value, ecological resilience across diverse habitats, and high concentrations of health-promoting nutrients. Historically, *Camassia* bulbs served as a vital food source and trade commodity among Indigenous communities, prized for their pleasant flavour, long shelf life, and nutritional richness [1,2,3,4,5,6,7]. Compared with conventional staple crops such as potatoes and beans, boiled *Camassia* bulbs exhibit greater concentrations of proteins, carbohydrates, vitamins, minerals, and dietary fibre [8,9,10].

The genus is characterized by relatively low cultivation requirements, thriving in full sun or light shade with consistent soil moisture from autumn until dormancy. Ecologically, *Camassia* occupies vernal meadows, riparian zones, and moist montane habitats, favouring soils with high spring moisture and good drainage [11,12]. *C. leichtlinii* (Baker) S. Watson demonstrates remarkable cold hardiness, tolerance to a wide range of soil textures, and minimal susceptibility to pests and pathogens, making it suitable for both ornamental cultivation and ecological restoration [1,6]. However, excessive moisture during dormancy can damage bulb tissue and disrupt flowering phenology [6,12]. Morphologically, *C. leichtlinii* produce basal linear–lanceolate foliage with a distinct central vein and a waxy cuticular layer that limits transpiration. Its bulbs, enclosed in suberized olive-brown tunics, range from 1.5 to 3.9 cm in diameter, reflecting adaptation to local edaphic and climatic conditions [13]. The racemose inflorescences bear actinomorphic, six-tepaled flowers in shades ranging from white and pale blue to deep violet. Although individual flowers remain open for approximately 24 h, sequential anthesis extends the blooming period over several weeks [8].

Despite its horticultural and ecological importance, *Camassia* remains insufficiently studied with respect to its physiological and metabolic responses to environmental fluctuations. The genus exhibits considerable metabolic plasticity, enabling modulation of reserve accumulation and secondary metabolite production under diverse cultivation regimes [3,5]. Understanding the biochemical adjustments is essential for optimizing cultivation practices and elucidating the molecular mechanisms underlying environmental adaptation in bulbous perennials. In this context, *Camassia* exemplifies the geophytic growth strategy, a survival form characterized by perennial species that persist through adverse seasons via underground storage organs such as bulbs, corms, and rhizomes [14,15,16]. These reserve structures function as repositories for carbohydrates, proteins, and minerals, ensuring regeneration and reproductive success when favourable conditions return [17,18]. The extent of vegetative and reproductive development correlates closely with the quantity and composition of these stored compounds [15,19,20]. Carbohydrates, particularly sucrose, dominate these stores, serving as energy sources, structural precursors, and key regulators of bulb enlargement and floral induction [17,21]. The regulation of carbohydrate metabolism and partitioning provides key insights into how plants coordinate growth, storage, and environmental adaptation [5]. The regulation of carbohydrate metabolism and partitioning is thus central to understanding plant growth, storage dynamics, and environmental adaptation. In addition to carbohydrates, amino acids, organic acids, and fatty acids contribute to energy balance and stress resilience. Amino acids provide nitrogen, regulate osmotic balance, and mediate stress signalling, particularly γ-aminobutyric acid (GABA) and proline, which accumulate under heat, drought, and salinity stress [22]. Organic acids sustain the tricarboxylic acid (TCA) cycle and respiration, thereby maintaining ATP production during stress and helping restore redox and energy homeostasis when respiratory flux is constrained [23]. Fatty acids, meanwhile, are crucial for membrane structure and function, as they preserve membrane integrity and participate in lipid-based signalling pathways that mediate abiotic stress responses [24]. Temperature is a key abiotic factor influencing bulb development and flowering. As a spring-flowering geophyte, *Camassia* requires a specific “warm–cold–warm” temperature sequence to complete its annual developmental cycle, with the cold (vernalization) phase being essential for floral induction [18,25]. Insufficient chilling can disrupt this process, leading to delayed emergence, impaired floral differentiation, and reduced flowering performance [15].

Recent advances in gas chromatography–mass spectrometry (GC–MS) have enabled comprehensive profiling of primary and secondary metabolites in bulbous and geophytic plants. GC–MS-based metabolomics facilitates the identification and quantification of soluble sugars, amino acids, and organic acids, offering detailed insights into metabolic dynamics under varying environmental and cultivation conditions [26]. It also detects selected low-molecular-weight phenolic compounds that can be volatilized through derivatization [27]. This approach is instrumental in linking biochemical traits to growth performance and in uncovering the molecular basis of plant adaptive strategies.

The present study was conducted in the northeastern region of Romania (47°9′44″ N, 27°35′20″ E), characterized by a temperate continental climate similar to those found in other temperate European landscapes, thereby enhancing the regional relevance and broader applicability of the findings. This investigation provides a comparative assessment of growth characteristics and metabolomic profiles, derived via GC-MS, in *Camassia leichtlinii* cultivars ‘Alba’ and ‘Caerulea’ grown under three distinct environmental conditions: open field, outdoor containers, and controlled greenhouse settings. The primary objective of this study is to promote the cultivation of this underutilized species in Romania, where it remains largely unrecognized despite its established ornamental and nutritional significance within its native North American range. The findings contribute novel insights into the adaptive strategies of bulbous perennials and advance the understanding and cultivation of *Camassia* species for both ornamental horticulture and nutritional applications.

## 2. Results

### 2.1. Morphological and Phenological Traits

**Figure 1:**
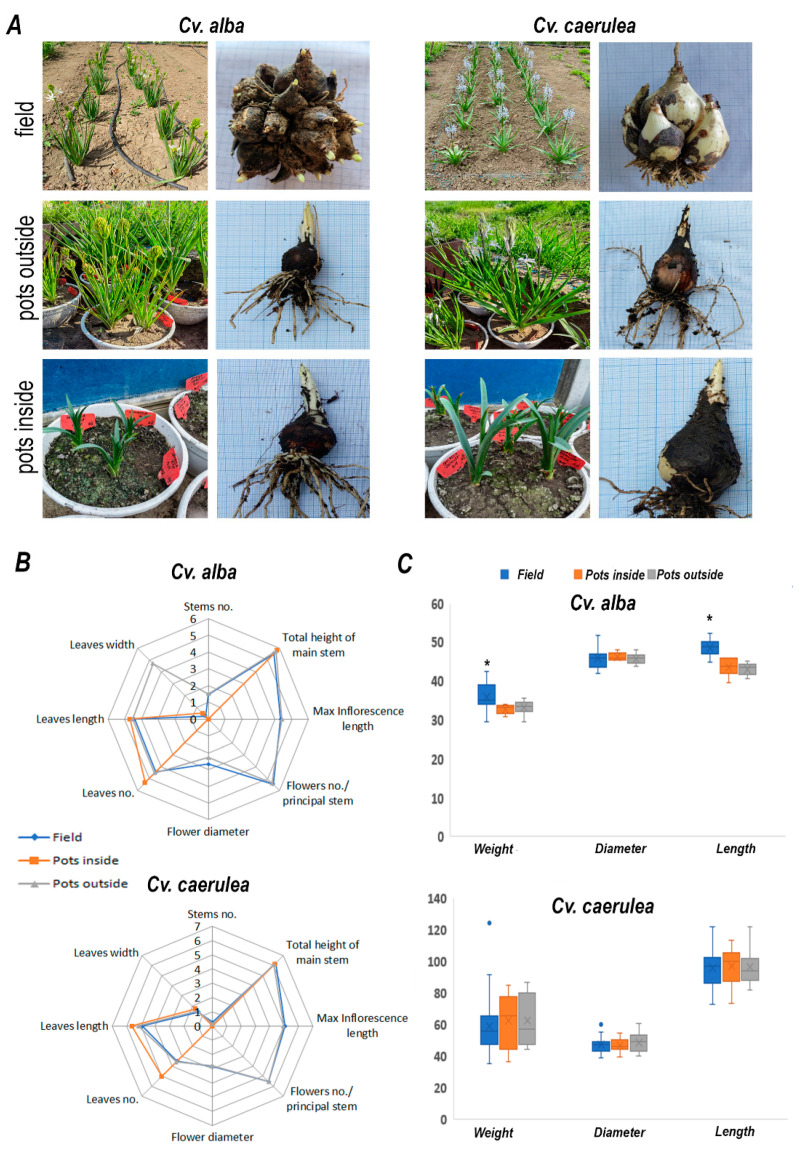
Plant material and phenotypes growth in different conditions. The left panel represents the vegetative part of plants, and the right panel represents bulbs. (**A**) cv. ‘Alba’ and cv. ‘Caerulea’ is grown in the field, pots inside, and pots outside. (**B**) Measurement of several phenotypic traits of cv. ‘Alba’ and cv. ‘Caerulea’ grown in different conditions is shown in the legend. (**C**) Bulb weight (g), diameter (mm), and length (mm) in plants grown in different conditions. Data represent mean ± standard deviation (*n* = 6–30, biological replicates per treatment). The statistical significance of the differences in bulb morphology was determined by ANOVA (* *p* < 0.05).

To thoroughly assess the impact of different cultivation systems on the growth and metabolic profile of two *Camassia* cultivars, experimental plots were established under three distinct conditions: open field, outdoor potted, and greenhouse potted environments (Figure 1). These culture systems were selected to simulate a range of environmental conditions, from natural field exposure to semi-controlled (outdoor pots) and fully controlled (greenhouse) settings, thereby enabling a comprehensive evaluation of environmental influence on plant development. Regarding the expression of morpho-decorative traits, it was observed that both cultivars responded distinctly to the cultivation environment. Notably, plants grown in pots under greenhouse conditions failed to initiate or develop flowering stems, regardless of cultivar (Figure 1A). However, we did not find any further differences concerning the number of leaves and size, and stem size, although the growth conditions slightly affected the number of leaves in cv. ‘Caerulea’, which developed more leaves in pots in greenhouse conditions (Figure 1B).

This suggests that while the greenhouse environment offers controlled temperature and protection from external stressors, it may inadvertently suppress floral induction cues, potentially due to reduced light intensity, altered photoperiods, or limited root volume. Biometric measurements of the bulbs were recorded both before planting and after two consecutive growing seasons. The parameters analyzed included bulb mass, diameter, and height (Figure 1C). To account for the potential risk of flowering suppression in subsequent years, a phenomenon occasionally observed in some rustic geophyte species, the bulbs were left undisturbed in their growing environment for the entire two-year period.

Following this period, notable differences were observed between the two *Camassia* cultivars. Cv. ‘Alba’ formed dense colonies consisting of 18–20 bublets per original plant, and the average mass of the colony was nearly doubled compared to that of the initial mother bulb. However, the largest bublet reached only about half the original bulb’s diameter and height. In contrast, cv. ‘Caerulea’ exhibited a different growth pattern, producing only 2–3 bublets per plant. Still, the colonies displayed a threefold increase in mass over the initial weight of the mother bulbs, indicating substantial development. Table 1 presents the key phenological data recorded for the 2023–2024 experimental cultures. It was observed that plants overwintered in the greenhouse initiated vegetative growth approximately two months after they were planted. These plants did not enter a dormancy phase and maintained continuous vegetative development throughout the period. However, they failed to initiate flowering. Interestingly, although the potted plants overwintered outdoors were exposed to the same environmental conditions as the in-ground field specimens, their phenological development was delayed in both cultivars. Specifically, the onset of vegetative growth in potted plants was delayed by approximately one month, followed by an additional ±10-day delay in the progression of subsequent phenophases.

**Figure 2:**
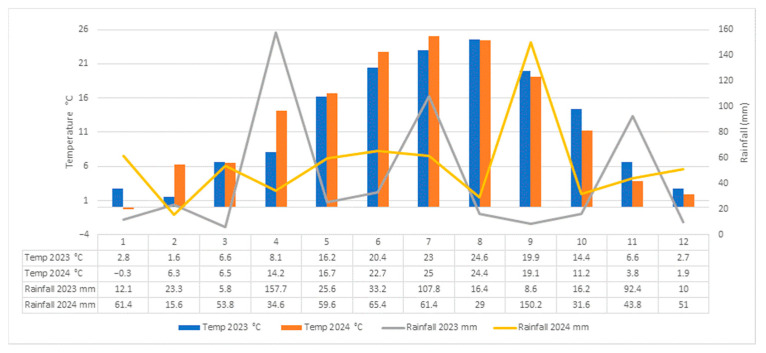
Average temperatures and monthly rainfall conditions in Iasi (Romania) in 2023–2024.

Interannual differences in field-grown plants were largely driven by the elevated temperatures recorded in 2024, which accelerated phenological development in both cultivars. Vegetative growth and flowering began approximately one week earlier than in the previous year, and peak flowering occurred about 14 days sooner. Despite these shifts in absolute timing, the intervals between successive phenophases remained largely unchanged, indicating that the cultivars preserved their intrinsic developmental rhythm. During the experimental years, temperature and humidity conditions varied considerably (Figure 2). Total monthly precipitation ranged from 5.8 mm in March to 157.7 mm in April, with the minimum and maximum values recorded in 2023. Average monthly temperatures ranged from −0.3 °C in January to 25 °C in July, the lowest and highest values being recorded in 2024. In 2023, precipitation showed pronounced fluctuations, with differences of up to 151 mm between months. Mean air temperatures remained positive throughout the year, ranging from 1.6 °C in February to 24.6 °C in August. In 2024, temperatures were slightly higher overall, with February and April being 5–6 °C warmer than in the previous year, which led to an earlier onset of vegetative growth. Precipitation in 2024 was more evenly distributed during the first part of the year but showed larger monthly differences between August and September (121 mm).

A noteworthy finding concerns the timing of first-flower anthesis and last-flower senescence. In potted plants, these stages occurred slightly later than in field-grown specimens; nevertheless, the total flowering period remained constant, 10 days for the first cultivar and 15 days for the second in the first experimental year. These results suggest that while environmental conditions (e.g., temperature or cultivation system) may influence phenological onset, the intrinsic duration of the flowering period is genetically fixed and cultivar-specific.

### 2.2. Estimation of Soil Water Balance and Irrigation Regime

**Figure 3:**
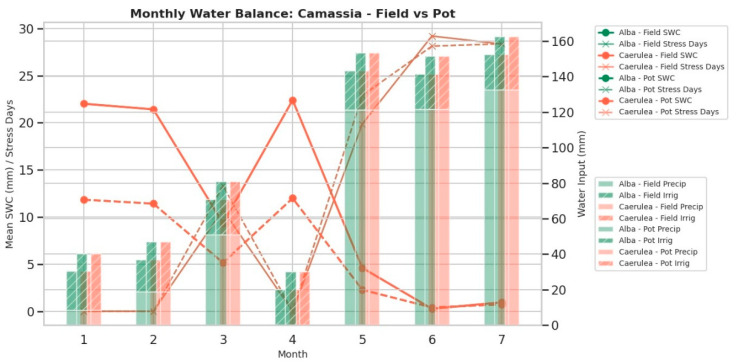
Monthly soil water content (SWC), days under water stress, and total precipitation for *Camassia alba* and *C. caerulea* under field conditions (2023).

Based on collected data, monthly derived agroclimatic parameters were calculated, including the sum of active temperatures (SumActT, °C·day), total sunshine duration (ASD_total, h), available energy index (IAOe, Tₐct × ASD_h), total precipitation (mm), mean evapotranspiration (ETc, mm·day^−1^), and soil water balance indicators such as number of stress days (days_stress), deficit_total (mm), and irrigation input (mm) [28]. The soil water balance was modelled separately for field and pot-grown *Camassia* plants, assuming daily variation in soil water content (ΔSWC = P + I − ETc). Hydric stress was considered when available soil water dropped below 50% of the calculated retention capacity (CRAS = 90 mm for field soils, 60 mm for pot (substrate) [29]. The aggregated data for the first two quarters (January–June), extended to July (Table 2), summarize the monthly and quarterly means ± SD for each growth condition and cultivar, while monthly dynamics of soil water content, stress days, and precipitation are illustrated in Figure 3. Results indicate that cumulative SumActT increased steadily from <20 °C·day in winter to >400 °C·day by July, while ASD_total exceeded 340 h in late spring–summer. Despite total precipitation exceeding 100 mm in May–July, evapotranspiration demand (ETc > 4 mm·day^−1^) led to persistent negative soil water balance, especially in field-grown plants (deficit >100 mm; 28–30 stress days per month).

Legend (abbreviations): Sum of active temperatures (SumActT, °C·day), total available energy index (IAOe_total), total precipitation (Total P, mm), number of days under water stress (Days_stress), cumulative soil water deficit (Deficit, mm), and total irrigation applied (Irrigation, mm). T1 corresponds to the pre-irrigation period (0 mm applied), while T2 corresponds to the irrigation period (Field: 3–4 days, 2 mm per event; Pot: 2 days, 2 mm per event).

Pot-grown plants showed smaller water deficits due to more frequent irrigation but exhibited higher variability in water status, reflecting the limited storage capacity of the substrate. These trends confirm that the second quarter (April–June, extended to July) represents a period of moderate to high hydric stress, providing a quantitative basis for correlating metabolite accumulation (e.g., organic acids, fatty acids) with drought-related physiological adjustments.

### 2.3. GC-MS Metabolite Profiles

The metabolite profiles of *Camassia* cultivars revealed coordinated adjustments influenced by genotype, environmental conditions, and developmental stage. Thirty-eight metabolites—including amino acids, organic acids, soluble sugars, sugar alcohols, and lipid derivatives were identified and quantified in Table S1 (page 2) of the Supplementary Materials. Total free amino acids, although present at low concentrations (0.25–0.95 mg g^−1^ DW), were consistently higher in cv. ‘Caerulea’, particularly in pot-grown plants, suggests increased nitrogen turnover and stress-induced protein catabolism in response to restricted soil volume and fluctuating moisture availability. In contrast to early field stages, total inorganic and organic acids increased in the 2-year field plants of both cultivars, with the most pronounced accumulation observed in cv. ‘Caerulea’. This trend indicates a stimulation of acid metabolism during prolonged field exposure, possibly reflecting intensified respiration and enhanced flux through the TCA cycle to meet energy demands and maintain redox balance in maturing tissues. Outdoor pot-grown plants also maintained elevated acid contents, consistent with their exposure to greater thermal and hydric variability. Mono- and disaccharides dominated the metabolite composition (30.09–44.60 mg g^−1^ DW) and were most abundant in cv. ‘Caerulea’, particularly under outdoor and 2-year field conditions (CC PO and CC 2YF). The elevated sugar levels point to reinforced carbon assimilation and storage capacity, underpinning improved energy reserves and osmotic regulation. Total sugar alcohols and related organic acids (3.77–5.55 mg g^−1^ DW) were moderately abundant and more pronounced in ‘Caerulea’, supporting their proposed roles as osmoprotectants and antioxidants contributing to stress tolerance.

Total fatty alcohols and alkanes (1.75–3.21 mg g^−1^ DW), together with total fatty acids (6.59–10.15 mg g^−1^ DW), increased progressively across both species and growth conditions, suggesting membrane remodelling and the activation of lipid-based stress responses. Collectively, these findings highlight cultivar-specific metabolic flexibility, with cv. ‘Caerulea’ exhibited more dynamic adjustments in carbohydrate, organic acid, and lipid metabolism, indicative of superior physiological resilience under diverse cultivation environments.

The diverse metabolite profile highlights the bulbs’ functions in energy storage, stress adaptation, and metabolic regulation, providing valuable insight into the physiological differences between cultivars and their responses to various cultivation systems (Table S1 (page 1) of the Supplementary Materials).

This interpretation is supported by the pedoclimatic monitoring and soil water balance modelling, which showed that cumulative active temperatures (SumActT) increased steadily from winter to mid-summer, while evapotranspiration demand (ETc) exceeded precipitation during April–July, which generated intermittent hydric stress (deficit >100 mm; 16–17 stress days per month in field-grown plants).

Pot-grown plants, even subjected to more frequent irrigation, exhibited higher variability in water status and reflected limited substrate water retention.

These environmental dynamics correlate with the observed metabolic adjustments in ‘Caerulea’, including increased accumulation of carbohydrates, organic acids, and lipids and confirm that cultivar-specific metabolic flexibility is closely linked to the capacity to cope with variable water availability and energy demands under different cultivation systems.

**Figure 4:**
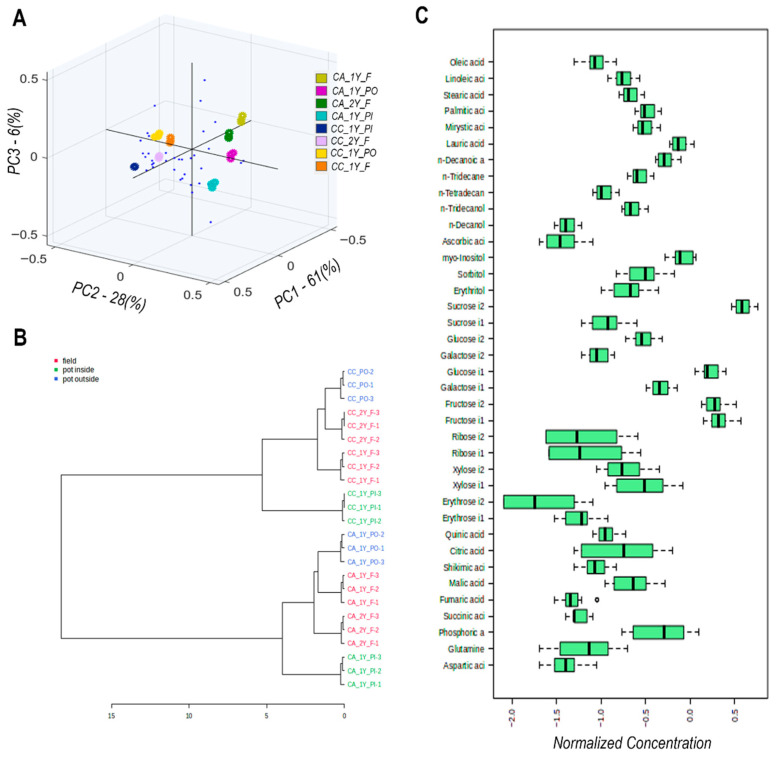
GC/MS metabolic profiling of bulbs from cv. ‘Alba’ and cv. ‘Caerulea’, grown in the field, pots inside, and pots outside for one and two years. (**A**) PCA biplot of the distribution of samples and identified metabolites in the three main PC coordinates. Loadings are represented with blue dots; scores are represented with ovals with different colours according to the colour bar. (**B**) HCA of samples according to metabolite content. (**C**) Bar charts of normalized variance of identified metabolites across samples. Abbreviations of samples are as follows: CA–*C*. ‘Alba’, CC–*C*. ‘Caerulea’, 1Y—one year, 2Y—two years, F—field, PO—pots outside, PI—pots inside.

Figure 4A presents a principal component analysis (PCA) of the metabolic profiles obtained from methanol extracts of *Camassia* bulbs, analyzed by gas chromatography-mass spectrometry (GC-MS). The PCA explains approximately 95% of the total variation across all samples, with the three principal components (PCs) offering insights into the main sources of metabolic variability (Table S1 (page 3) of the Supplementary Materials). PC1, accounting for 61% of the variation, reflects the influence of environmental conditions by clearly separating field-grown samples (F) from those cultivated in indoor pots (PI). In contrast, outdoor pots (PO) occupy an intermediate position. PC2, which accounts for 28% of the variation, reflects genetic differences between the two cultivars, ‘Alba’ and ‘Caerulea’, as evidenced by their opposing distribution along the axis, indicating a fundamental divergence in metabolic expression. PC3, explaining 6% of the total variation, captures age-related differences, with two-year-old samples (2Y) showing a slight orthogonal shift from one-year-old samples (1Y), suggesting more subtle maturity-related metabolic changes. Key metabolites contributing to the PCA distribution include fructose, fumaric acid, n-decanoic acid, myristic acid, n-tetradecanol, sucrose, linoleic acid, and quinic acid (positive loadings on PC1), as well as phosphoric acid and erythrose (negative loadings on PC1). Ascorbic acid, xylose, glutamine, and ribose contributed primarily to the negative loadings of PC2. These findings highlight environmental factors as the dominant driver of metabolic variation, followed by genetic background, while plant age plays a secondary role.

The hierarchical clustering dendrogram (Figure 4B) illustrates the metabolic similarity among samples based on Euclidean distance and the complete linkage method. Field-grown specimens (F) form a cohesive and distinct cluster, indicating a consistent metabolic response under open-field conditions. In contrast, indoor pot-grown samples (PI) also cluster tightly together, reflecting the uniformity imposed by controlled environmental parameters. Outdoor pot samples (PO), however, are distributed across two subclusters, suggesting heterogeneous metabolic responses likely due to variable abiotic stresses encountered in semi-controlled outdoor conditions. This hierarchical structure reinforces the conclusion that the cultivation environment is the primary determinant of metabolic patterns, while genetic differences between cultivars introduce secondary stratification within each main cluster.

Boxplots comparing the distribution of normalized relative abundances of representative metabolites, including fatty acids (oleic, linoleic, palmitic), sugars (glucose, fructose, sucrose), and organic acids (malic, citric, fumaric), reveal distinct biochemical responses across growing conditions and cultivars in agreement with their contribution in PC scales (Figure 4C).

Cultivar-specific differences are evident, as cv. ‘Alba’ accumulates significantly higher levels of sucrose, whereas cv. ‘Caerulea’ exhibits elevated glucose concentrations, indicating intrinsic genetic divergence in carbohydrate storage and mobilization strategies. These results emphasize the role of osmoprotective and energy-regulating compounds in stress response and help delineate distinct metabolic signatures associated with both cultivation systems and genotypic background.

**Figure 5:**
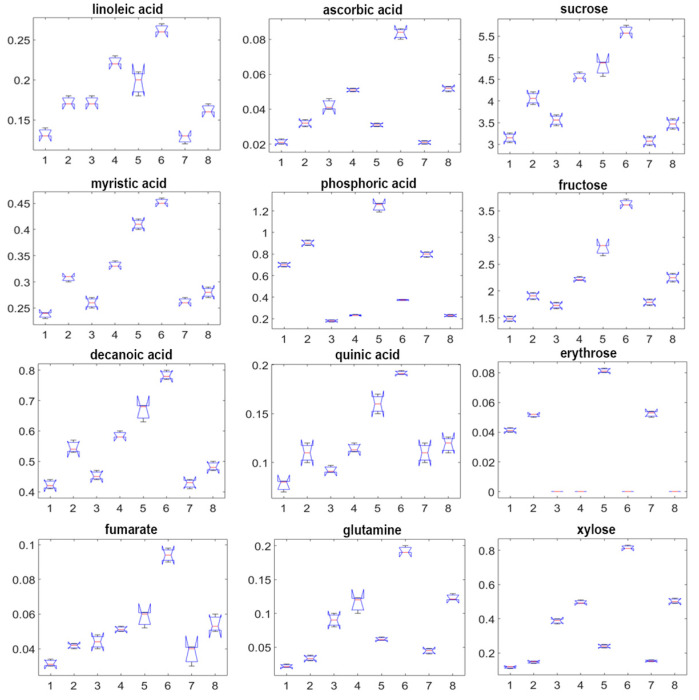
Changes in metabolites with the highest contribution in the PCA plot, cv. ‘Alba’ and cv. ‘Caerulea’, grown in the field, pots inside, and pots outside for one and two years. X axes represent samples as follows: 1-CA_1Y_F, 2-CA_2Y_F, 3-CC_1Y_F, 4-CC_2Y_F, 5-CA_1Y_PI, 6-CC_1Y_PI, 7-CA_1Y_PO, 8-CC_PO; Y axes represent the averaged concentration of metabolites normalized to the internal standard. Abbreviations of samples are as follows: CA—*C.* ‘Alba’, CC—*C.* ‘Caerulea’, 1Y—one year, 2Y—two years, F—field, PO—pots outside, PI—pots inside.

The box-and-whisker diagram (Figure 5) illustrates the specific metabolites exhibiting the greatest variation across samples with their relative concentration distributions. Four principal metabolite classes-polyunsaturated fatty acids, soluble carbohydrates, organic acids, and free amino acids-across eight distinct plant samples (1-CA_1Y_F, 2-CA_2Y_F, 3-CC_1Y_F, 4-CC_2Y_F, 5-CA_1Y_PI, 6-CC_1Y_PI, 7-CA_1Y_PO, and 8-CC_PO) were represented. Fructose, n-decanoic acid, myristic acid, fumaric acid, n-tetradecanol, sucrose, linoleic acid, and quinic acid all show significantly elevated levels in bulbs cultivated in indoor pots for both cultivars, with cv. ‘Alba’ consistently accumulates higher concentrations than cv. ‘Caerulea’. Additionally, ascorbic acid, xylose, glutamine, and ribose were significantly more abundant in cv. ‘Alba’ across all cultivation conditions, with the highest levels recorded in indoor potted samples. These findings emphasize the combined effects of genotype and environmental conditions on metabolite accumulation, offering insight into cultivar-specific stress responses and adaptive mechanisms.

Figure samples exhibit the highest levels of oleic and malic acids; both associated with cellular defence and abiotic stress adaptation mechanisms. In contrast, indoor-cultivated plants display notably reduced concentrations of sugars and unsaturated fatty acids, reflecting lower energy demand and a downregulation of lipid metabolism typical of growth under optimal, stress-free conditions.

### 2.4. Pathway Analysis

**Figure 6:**
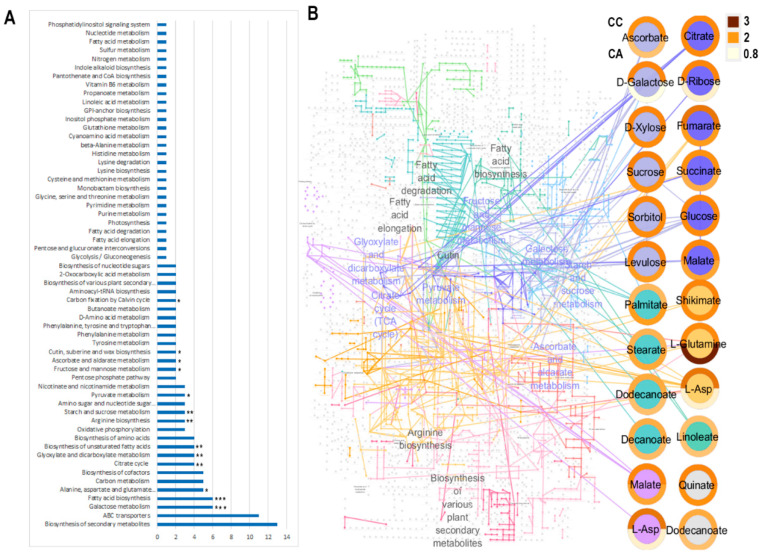
Pathway analysis. (**A**) KEGG mapping of identified metabolites. Metabolic pathways (y-axis) with more than two mapped metabolites are represented with the corresponding number of mapped metabolites (x-axis). The statistical significance of enrichment of metabolites for each pathway is given by an asterisk (* *p* < 0.05; ** *p* < 0.01; *** *p* < 0.001). (**B**) Reconstruction of KEGG metabolic pathways. Identified metabolites are represented as coloured nodes for different metabolite pathways assigned in the map; donut diagrams around each node represent the fold change ratio between plants grown in the field and in pots inside, according to the colour bar. The upper part of the diagram corresponds to cv. ‘Caerulea’ and the lower part to cv. ‘Alba’ is shown for the upper node in the left column.

The identified metabolites were enriched in multiple metabolic pathways according to the KEGG database, including biosynthesis of secondary metabolites, ABC transporters, galactose metabolism, fatty acid biosynthesis, alanine, aspartate and glutamate metabolism, carbon metabolism, biosynthesis of cofactors, the citrate cycle, glyoxylate and dicarboxylate metabolism, biosynthesis of unsaturated fatty acids, biosynthesis of amino acids, and oxidative phosphorylation (Figure 6A).

The significantly enriched pathways-galactose, fatty acid biosynthesis, citrate and glyoxylate cycles, sucrose and starch metabolism and amino acid metabolism indicate a coordinated reprogramming of lipid, sugar, organic acid and amino acid metabolism, which are critical for maintaining growth and accumulation of primary and secondary metabolites (Figure 6A). Core carbon metabolites, including glucose, sucrose, malate, and L-aspartate, emerge as central hubs underscoring their pivotal roles in distributing carbon skeletons, fueling the tricarboxylic acid (TCA) cycle.

Overall, nearly all identified metabolites exhibited higher concentrations in bulbs from plants grown in indoor pots for both species (Figure 6B). Galactose, aspartate, and ribose accumulated more prominently in cv. ‘Caerulea’ under indoor pot conditions compared to the cv. ‘Alba’, whereas glutamine showed greater accumulation in cv. ‘Alba’ grows indoors compared to the cv. ‘Caerulea’.

## 3. Discussion

Environmental conditions emerged as the primary determinants of both morphological and metabolic variation in *Camassia* sp., particularly under greenhouse cultivation. Phenological data revealed a marked delay in vegetative growth and floral initiation in plants grown in outdoor pots (PO) compared to those cultivated in the field, whereas indoor (greenhouse) plants (PI) exhibited continuous vegetative growth without flowering (Figure 1A, B). The complete suppression of flowering in PI plants indicates a disruption of photoperiodic or temperature-responsive signalling pathways. Although the greenhouse environment provides controlled temperature, reduced water stress, and protection from external biotic and abiotic challenges, it may inadvertently inhibit floral induction cues. This suppression is likely linked to lower light intensity, altered photoperiod regimes, or restricted root volume, all of which can modulate hormonal and carbohydrate signalling networks involved in flowering induction [30,31]. These findings emphasize the profound influence of cultivation context on the balance between vegetative persistence and reproductive competence in *Camassia*, providing important insights for ornamental breeders targeting year-round flowering and optimized horticultural performance. Notably, bulbs of both *Camassia* species exhibited greater mass and diameter under field conditions compared to those grown in pots, suggesting that unrestricted soil volume and natural environmental cues promote more robust underground storage organ development. In contrast, greenhouse-grown bulbs remained smaller and less differentiated, likely due to spatial constraints and altered resource allocation under controlled conditions.

Previous analyses of *Camassia* sp. bulbs collected from regions including Victoria (Southwest coast of Vancouver Island) as well as Utah, Oregon, Washington, Montana, and Idaho, have described a nutritional profile characterized by low lipid and protein contents with a predominance of carbohydrates. These carbohydrates mainly comprise inulin-type fructans, dietary fibers, neutral polysaccharides, and osmoprotective mucilages. The bulbs also contain essential macronutrients-potassium (K), phosphorus (P), calcium (Ca), magnesium (Mg), and coenzymatic trace elements such as iron (Fe), zinc (Zn), copper (Cu), and manganese (Mn), alongside water-soluble B-vitamins and ascorbic acid, which collectively support cellular metabolism and antioxidant defenses. Moreover, the presence of phenolic compounds (flavonoids, hydroxycinnamic acids), saponins, and plant lectins contributes notable anti-inflammatory and immunomodulatory activities, underpinning their traditional dietary and medicinal uses [1,12,25,32].

GC–MS profiling revealed a diverse spectrum of primary and secondary metabolites, reflecting both environmental modulation and genotypic specificity. Quantitative comparison of field and potted (outdoor and indoor) samples showed that field-grown plants accumulated higher levels of soluble sugars and unsaturated fatty acids, consistent with enhanced photosynthetic flux and abiotic stress acclimation. Conversely, greenhouse-grown (PI) plants exhibited lower sugar and lipid concentrations, indicative of a more stable but metabolically subdued physiological state.

Notably, the accumulation of glutamine and sucrose in cultivar ‘Alba’ suggests superior nitrogen assimilation efficiency and osmoprotection capacity, aligning with its vigorous bulblet formation. In contrast, cv. ‘Caerulea’ displayed a metabolic bias toward biomass consolidation within the mother bulb rather than vegetative propagation.

Metabolic network analysis confirmed that primary carbon and nitrogen metabolism orchestrate the global metabolic response to environmental variation, integrating carbohydrate partitioning with amino acid biosynthesis. This coordinated regulation underscores *Camassia*’s potential as a model for studying stress physiology, storage organ metabolism, and the ecological adaptation of geophytes. These findings highlight environmental factors as the dominant driver of metabolic variation, followed by genetic background, while plant age plays a secondary role. Polyunsaturated fatty acids, especially linoleic acid, were consistently abundant across samples and are pivotal for maintaining membrane fluidity and serving as precursors to oxylipin-derived phytohormones involved in abiotic stress signalling. The narrow interquartile ranges and stable medians observed in GC–MS boxplots reflect a tightly regulated lipid biosynthetic network, facilitating rapid adjustment to temperature and humidity fluctuations. Simple sugars such as fructose, erythrose, xylose, and the disaccharide sucrose serve dual functions as immediate carbon and energy sources, as well as osmoprotectants that regulate cellular water potential. The pronounced variability in carbohydrate levels among samples reflects genotypic differences in carbohydrate storage and remobilization capacity, with direct implications for drought resilience and photosynthetic efficiency in cultivated *Camassia* taxa. Compared with the bulb carbohydrate data reported by [33], which showed total non-structural carbohydrates of 48.6 mg/g DW, our results are in close accordance, confirming similar levels of soluble carbohydrate accumulation. Acids, particularly phosphoric acid (a vital component of ATP), malic acid, and quinic acid, are central to respiratory metabolism and serve as substrates for the synthesis of defense-related secondary metabolites. The concentration fluctuations captured in the boxplots reveal dynamic regulation of the tricarboxylic acid (TCA) cycle and phenylpropanoid pathway in response to nutrient availability and environmental stresses, contributing to redox homeostasis and antioxidant metabolite accumulation. Free amino acids, particularly glutamine and asparagine, played central roles in nitrogen transport and assimilation. The broad interquartile range of glutamine concentrations reflects substantial metabolic flexibility, supporting biomass accumulation and tissue regeneration under variable cultivation regimes [34].

Food composition analyses of underground organs from lupin (*Lupinus* spp.), rice root (*Fritillaria camschatcensis*), licorice fern (*Polypodium glycyrrhiza*), wood fern (*Dryopteris* spp.), arrow leaf balsamroot (*Balsamorhiza sagittata*), bitterroot (*Lewisia rediviva*), common camas (*Camassia quamash*), cous biscuitroot (*Lomatium cous*) revealed the following average compositional ranges: moisture 66–81%, crude protein 0.6–3.1%, crude lipid 0.3–4.6%, and ash 0.7–1.0%. The energy value is between 71 and 138 kcal per 100 g, comparable to that of boiled potatoes, although the calcium content (31–84 mg/100 g) significantly exceeds the level in tubers (≈7 mg/100 g). Major minerals and trace elements included sodium (1–123 mg/100 g), iron (0.8–10 mg/100 g), zinc and copper (0.2–1.5 mg/100 g), while manganese, strontium, and the vitamin profile are found in low concentrations. Comprehensive compositional profiling establishes a fundamental framework for elucidating carbohydrate metabolism and evaluating the nutritional potential of geophytic species [35,36]. Future research should investigate whether the suppression of flowering observed under confined conditions is reversible. Replanting bulbs previously grown in restricted environments into open soil may help clarify this question and offer valuable insights into the physiological and developmental plasticity of the species. Such studies could further elucidate the relative roles of environmental and endogenous factors in regulating floral induction and reproductive success.

## 4. Materials and Methods

### 4.1. Plant Material and Cultivation Conditions

This study was conducted in the NE area of Romania (47°9′44″ N 27°35′20″ E), under temperate continental climate conditions, on two horticultural selections of *Camassia leichtlinii* (Baker) S. Watson. Cultivar ‘Alba’, characterized by cream-white to bluish-white flowers with elongated purple stamens, while ‘Caerulea’ produces tall stems topped by large pale-blue flowers with violet undersides and long yellow anthers.

Plants were monitored weekly, and no symptoms of pathogen infection were observed.

The experimental cultures were established in the last week of October 2022 at the Floriculture Department of the Iași University of Life Sciences, Romania, using both open-field and 4L pots systems. The garden soil used in the pots was identical in composition to the field substrate. Detailed physicochemical characteristics of the soil are provided in Table 3.

Healthy bulbs, relatively uniform in size and weight, were used to establish the crops. The average weight of ‘Alba’ and ‘Caerulea’ bulbs before planting was 33.6 g and 60.7 g, respectively. The average diameters were 42.7 mm and 47.8 mm, and the average lengths were 46.8 mm and 97.3 mm for ‘Alba’ and ‘Caerulea’, respectively. The potted plants were divided into two overwintering regimes: greenhouse and outdoor. Plants in the warm greenhouse were maintained at an average temperature between 20 and 26 °C year-round, with relative humidity ranging from 60 to 75%. Samples were harvested from one- and two-year-old field-grown plants, while potted bulbs were collected after a single growth season.

### 4.2. Phenological and Biometric Observation

Phenophases, including the onset of vegetative growth, floral stem emergence, flowering dates, and dormancy, were recorded for each treatment group over the 2023 growing season. All bulbs were initially similar in size and mass. Plants were monitored over two vegetation periods, and biometric data were recorded before planting and at the end of each growing season. The morpho-decorative characters tracked were bulb weight, height, and diameter before and after two consecutive growing seasons; number of the flower stems and the total height from the ground to the top of the plant, maximum length of the inflorescence and number of flowers on the principal stem; number of leaves, leaf length and width. Additionally, the phenophases were recorded for the 2024 growing season for the field-grown plants. All bulbs used in the study were initially uniform in size and mass to ensure consistency in the experimental material.

### 4.3. GC-MS Metabolite Profiling

The extraction of polar and lipid fractions followed the protocols of [37,38], and our previous works [26,39] with slight modifications: Briefly, 50.0 mg of the lyophilized sample (in three replicates) was mixed with 500.0 µL of methanol, 50.0 µL of ribitol, and 50.0 µL of n-nonadecanoic acid (internal standards, 1 mg/mL in methanol for polar and non-polar metabolite quantification, respectively). The mixture was incubated at 70 °C and 300 rpm for 30 min on a Thermo Shaker TS-100 (Analytik Jena AG, Jena, Germany). After cooling to room temperature, 200.0 µL water and 500.0 µL chloroform were added, and the sample was centrifuged at 13,000 rpm for 5 min at 22 °C (Beckman Coulter, Brea, CA, USA). Two fractions were collected and vacuum-dried at 40 °C using a centrifugal concentrator (Labconco Centrivap, Labconco Corporation, Kansas City, MO, USA): (i) upper phase, 300 µL (amino acids, organic acids, and sugars); (ii) lower phase, 300 µL (fatty acids). Derivatization: (i) the dried residue was dissolved in 300 µL methoxyamine hydrochloride (20 mg/mL in pyridine) and incubated at 90 °C and 300 rpm for 1 h. Subsequently, 100 µL N, O-Bis(trimethylsilyl)trifluoroacetamide (BSTFA) was added, and the mixture was heated at 75 °C and 300 rpm for 1 h. A 1 µL aliquot was injected into the GC–MS; (ii) the dried residue was treated with 1.0 mL 2% H_2_SO_4_ in methanol and incubated at 96 °C and 300 rpm for 1 h. Following three extractions with 10 mL n-hexane, the combined organic layers were vacuum-dried. The residue was derivatized with 100 µL pyridine and 100 µL BSTFA at 75 °C and 300 rpm for 40 min before GC–MS analysis. The phytochemical composition of *Camassia* bulb extracts was analyzed using an Agilent 7890A gas chromatograph coupled to a 5975C mass-selective detector (Agilent Technologies, Santa Clara, CA, USA). Separation was performed on a 30 m × 0.25 mm i.d. DB-5ms fused silica column with a 0.25 µm poly(dimethylsiloxane) stationary phase, using helium as the carrier gas at a flow rate of 1.0 mL/min. Sample injection (1 µL) was conducted in split mode (10:1) with the injector and transfer line maintained at 250 °C. For polar metabolites, the oven temperature was programmed to increase from 100 °C (2 min) to 180 °C at 15 °C/min, then to 300 °C at 5 °C/min, and held for 10 min. Lipids were analyzed with an initial temperature of 60 °C (2 min) ramped to 300 °C at 5 °C/min, and held for 10 min. The mass spectrometer operated in electron impact (EI) mode at 70 eV, scanning 50–550 *m*/*z*. Retention indices (RIs) were determined using a C10–C40 n-alkane mixture (Sigma) and calculated with AMDIS software (v2.73, NIST). Compounds were identified by comparing RIs and mass spectra against a custom reference library, the Golm Metabolome Database (accessed 6 March 2025) [40], and the NIST’08 Mass Spectral Library [41].

### 4.4. Statistical and Pathway Analysis

Metabolite data were subjected to principal component analysis (PCA) and hierarchical clustering (HCA). The imputed data matrix contained both species with all field trials and respective replicates (n = 3) as rows and the normalized peak area of identified metabolites as columns. Before analyses, the data matrices were log2-transformed and standardized to a zero mean and unit variance. All data were analyzed with MATLAB (MathWorks, MA, USA; R2015a software according to standard procedures. All identified metabolites were functionally mapped in the KEGG database using the available online mapping tool in the database. Further, pathway files enriched with metabolites were downloaded and condensed with Cytoscape v.3.7.1software [42]. The Omics analyzer plugin was used to visualize the log2-transformed averaged fold changes in metabolites between plants grown in pots inside and outside for both species. One-way ANOVA was used for statistical significance.

## 5. Conclusions

This study provides a comprehensive, multi-dimensional insight into how cultivation environment and genotype shape the growth and metabolic physiology of *Camassia leichtlinii*. Our integrated analysis of morphological, phenological, and metabolomic data demonstrates that environmental conditions are the primary driver of metabolic variation (61%), followed by genotypic differences (28%) and plant age (6%).

Statistical analyses of GC–MS data clearly delineated the environmental impact. Hierarchical Clustering Analysis (HCA) showed that field-grown (F) and indoor pot-grown (PI) plants formed distinct, tight clusters, whereas outdoor pot (PO) samples displayed heterogeneous metabolic responses, mirroring their exposure to variable abiotic stresses. The controlled greenhouse (PI) environment promoted a state of accumulation, significantly enriching primary metabolites like fructose, fumaric acid, and sucrose in cv. ‘Alba’. In stark contrast, field-grown plants, subjected to quantifiable hydric stress (soil water deficit > 100 mm; 16–17 stress days per month), exhibited adaptive signatures characterized by elevated levels of oleic acid, malic acid, and unsaturated fatty acids, crucial for membrane remodelling, TCA cycle energy production, and stress signalling. Pot-grown plants further underscored this link, as their limited substrate retention led to variable water status and correlated with increased accumulation of carbohydrates and lipids in ‘Caerulea’.

Cultivar-specific strategies were clearly defined. Cv. ‘Alba’ consistently maintained high sucrose and glutamine levels, supporting efficient carbon storage and nitrogen assimilation for bulblet proliferation. Conversely, cv. ‘Caerulea’ demonstrated greater metabolic plasticity, enhancing its resilience and biomass through stress-induced biosynthesis of amino and organic acids. Pathway analysis corroborated these differences, revealing significant enrichment in galactose metabolism, fatty acid biosynthesis, and the citrate cycle.

In summary, this work reveals a fundamental trade-off: greenhouse cultivation optimizes vegetative growth and metabolite accumulation, while field conditions enhance metabolic diversity and stress resilience. These findings highlight the biochemical plasticity of *Camassia leichtlinii*, confirming its dual potential for ornamental and nutritional applications. By successfully cultivating and profiling the species in a temperate continental climate, this study strongly supports the introduction and regional relevance of this underutilized North American species into novel horticultural and ecological contexts, such as those in Romania.

## Figures and Tables

**Table 1:** The phenological data of *Camassia*.

Cv.	Cult. Sist.	Onset of Vegetative Growth	Emergence of the Floral Stem	Flowering		Entry into Dormancy
				Start	End	
Cv. ‘Alba’	field	3 March 2023	1 May 2023	26 May 2023	5 June 2023	18 July 2023
		19 February 2024	23 April 2024	12 May 2024	27 May 2024	9 July 2024
	pots out	26 March 2023	12 May 2023	2 June 2023	12 June 2023	24 July 2023
	pots in	18 December 2022	-	-	-	-
Cv. ‘Caerulea’	field	7 March 2023	17 April 2023	3 May 2023	18 May 2023	20 June 2023
		19 February 2024	9 April 2024	19 April 2024	2 May 2024	6 June 2024
	pots out	23 March 2023	4 May 2023	13 May 2023	28 May 2023	30 June 2023
	pots in	12 December 2022	-	-	-	-

**Table 2:** Quarterly pedoclimatic and soil water balance parameters for *Camassia* (2023).

Growth/Cultivar/T	SumActT (°C·d)	IAOe_Total	Total P (mm)	Days_Stress	Deficit (mm)	Irrigation (mm)
Field/Alba/T1	6.53 ± 9.96	18.76 ± 21.6	25.24 ± 26.4	3.6 ± 6.2	8.36 ± 9.3	0 ± 0
Field/Alba/T2	180.27 ± 170.1	185.89 ± 151.6	80.9 ± 69.7	16.6 ± 14.8	196.6 ± 155.4	21 ± 1
Field/Caerulea/T1	6.53 ± 9.96	18.76 ± 21.6	25.24 ± 26.4	3.6 ± 6.2	8.36 ± 9.3	0 ± 0
Field/Caerulea/T2	180.27 ± 170.1	185.89 ± 151.6	80.9 ± 69.7	16.6 ± 14.8	196.6 ± 155.4	21 ± 1
Pot/Alba/T1	6.53 ± 9.96	18.14 ± 20.0	25.24 ± 26.4	4.4 ± 7.7	8.36 ± 9.3	0 ± 0
Pot/Alba/T2	180.27 ± 170.1	159.38 ± 58.5	80.9 ± 69.7	17.0 ± 14.4	196.6 ± 155.4	31 ± 1
Pot/Caerulea/T1	6.53 ± 9.96	18.14 ± 20.0	25.24 ± 26.4	4.4 ± 7.7	8.36 ± 9.3	0 ± 0
Pot/Caerulea/T2	180.27 ± 170.1	159.38 ± 58.5	80.9 ± 69.7	17.0 ± 14.4	196.6 ± 155.4	31 ± 1

**Table 3:** Garden soil characteristics.

Parameters		Granulometric Analysis		Micro Elements, Accessible Form (ppm)	
pH	7.77	Coarse sand 2.0–0.2%	1.3	Zn	8.60
N%	0.15	Fine sand 0.2–0.002%	39.7	Cu	13.87
P (ppm)	268	Dust 0.02–0.002%	26.3	Mn	31.56
K (ppm)	230	Colloidal clay < 0.002%	32.7	Fe	10.49
Hummus%	3.83	Physical clay < 0.01%	44.1		
Organic matter%	4.86				

## Data Availability

The original contributions presented in this study are included within the article and the Supplementary Materials.

## Supplementary Materials

The following supporting information can be downloaded at: https://www.mdpi.com/article/10.3390/molecules30234520/s1, Table S1: Supplementary Materials.
